# Effects of Enclosure Size on the Preferences of Juvenile Chinchillas (*Chinchilla lanigera*)

**DOI:** 10.3390/ani15172483

**Published:** 2025-08-24

**Authors:** Zsolt Szendrő, Stanisław Łapiński, Zsolt Matics, Zsolt Gerencsér

**Affiliations:** 1 Institute of Physiology and Nutrition, Hungarian University of Agriculture and Life Sciences (Kaposvár Campus), Guba Sándor Str. 40, 7400 Kaposvár, Hungary; szendro.zsolt@uni-mate.hu; 2 Department of Zoology and Animal Welfare, University of Agriculture in Krakow, al. Mickiewicza 21, 31-120 Kraków, Poland; 3 Department of Animal Sciences, Széchenyi István University, 9200 Mosonmagyaróvár, Hungary; matics.zsolt@ga.sze.hu; 4 Institute of Animal Sciences, Kaposvár Campus, Hungarian University of Agriculture and Life Sciences, Guba Sándor Str. 40, 7400 Kaposvár, Hungary; gerencser.zsolt@uni-mate.hu

**Keywords:** *Chinchilla lanigera*, juvenile behavior, cage size preference, enclosure design, animal welfare

## Abstract

This study investigated the cage size preferences of juvenile chinchillas using enclosures that allowed free choice between different dimensions. Three comparisons were tested: (1) small floor area (0.15 m^2^) vs. double floor area (0.30 m^2^) at constant height; (2) low height (0.4 m) vs. high height (1.0 m) at constant floor area (0.15 m^2^); and (3) small–low cage (0.15 m^2^ × 0.4 m) vs. large–high cage (0.30 m^2^ × 1.0 m). The juveniles consistently preferred the smaller or lower enclosures across all trials. In the floor area tests, chinchillas spent about 66–75% of their time in the small cage compartments (*p* < 0.001). In the height comparison, the low cage was preferred by 70% (*p* < 0.001). When both floor area and height were increased, the small–low cage was preferred by 79% (*p* < 0.001). Differences in preference were most evident during the daytime period. At night, however, cage utilization was more even. These results indicate that young chinchillas strongly prefer smaller, lower spaces. Based on these preference tests alone, simply providing larger cages (without other modifications) did not result in greater use by juvenile chinchillas. These unexpected results suggest that factors such as safety or familiarity drive juveniles to choose smaller enclosures. Further research is needed to clarify the motivation behind these preferences and to determine how best to incorporate them into improved welfare-oriented housing designs.

## Simple Summary

Chinchillas are commonly kept for fur production and as pets, but little is known about their housing preferences. This study investigated whether young chinchillas prefer larger cages or smaller, more enclosed spaces. The animals were allowed to choose between cages with different floor areas, heights, or both, and their behavior was monitored over several days. Surprisingly, young chinchillas consistently preferred smaller and lower cages. These results suggest that young chinchillas prefer tight, sheltered spaces, likely because they feel safer. However, this does not mean that small cages are ideal for their welfare. Instead, the results emphasize the importance of providing safe hiding places in larger, more diverse environments. Understanding these preferences can help improve chinchilla housing by balancing their need for safety with space to move and explore, which ultimately promotes better animal welfare.

## 1. Introduction

*Chinchilla lanigera*, the long-tailed chinchilla, is a small South American rodent, native to the high Andes of northern Chile. Historically, this species ranged across parts of Bolivia, Peru, and Argentina, but intensive hunting for their luxuriantly dense fur drove them to near extinction in the wild by the late 19th century [1]. Today, remnant wild chinchilla colonies survive only in isolated arid mountain areas of north-central Chile, typically at elevations of 400–1650 m, with some reports up to 3000–5000 m [2,3]. Their natural habitat consists of rugged rocky slopes with sparse vegetation (thorny shrubs, cacti, bromeliads, and grasses) characteristic of the dry Andean foothills [4]. Chinchillas are well-adapted to these harsh conditions; the climate in their range can swing from 30 °C in the daytime down to below freezing at night. To survive such extremes, chinchillas have evolved an exceptionally dense, velvety fur (over 20,000 hairs per cm^2^) that provides superb insulation [1,4]. They escape daytime heat by sheltering in rock crevices or burrows and emerge mostly at dusk and during the night to forage [5]. This strict nocturnal and crepuscular activity pattern helps them avoid predators, which include birds of prey and various mammalian carnivores [6].

In the wild, chinchillas are gregarious and live in colonies sometimes exceeding 100 individuals [3]. Within these colonies, the population density can vary widely (0.9–10.7 animals per 10,000 m^2^) depending on habitat and resource availability [6]. Group living offers anti-predator benefits: with many eyes on watch, threats can be detected early, and a predator chasing multiple fleeing chinchillas will have difficulty singling out one target [7]. Chinchillas also rely on agility to evade predation. They have powerful hind legs that enable sudden sprints and standing jumps over 1 m high and 2 m long [7], allowing them to navigate rocky terrain swiftly. Besides these survival behaviors, wild chinchillas perform characteristic activities such as dust bathing in fine volcanic sand to maintain their plush coat [8]. Indeed, sandbathing helps remove excess oils from their fur [9]. They are generalist herbivores that nibble a variety of dry mountain plants; their diet includes grasses, herbs, and even succulent cactus pads [6].

The near disappearance of wild chinchillas due to fur hunting led to early efforts at captive breeding. A pivotal event was the rescue of a small founder population by Mathias Chapman in 1923, who brought 11 (or 12) wild chinchillas from Chile to California [10]. Virtually all domestic and farmed chinchillas worldwide today descend from this nucleus [10]. Over the past century, *C. lanigera* has been bred extensively in captivity, primarily for the fur industry and secondarily for use in biomedical research [11] or as a companion animal. By the 21st century, chinchilla farming had become well established, with hundreds of thousands of pelts produced annually. For example, around 200,000 chinchilla furs were sold in Europe in 2016 [12].

In modern farming, chinchillas are typically kept in indoor facilities under controlled climate conditions that protect them from extreme temperatures. Housing is usually in the form of tiered wire-mesh cages arranged in polygamous breeding units [13]. A common configuration is a 4:1 female-to-male ratio [13]. Standard cages used on commercial farms are relatively small, often around 0.5 m × 0.4 m × 0.4 m [14]. These dimensions are considerably smaller than some recommended guidelines; for instance, the Council of Europe has suggested a minimum enclosure floor area of 0.5 m^2^ per adult chinchilla with at least 1 m height [15]. Furthermore, there is currently no globally standardized regulation mandating cage size for chinchillas in farming contexts, and national regulations vary significantly or may be absent altogether in some countries.

The stark contrast between the wild chinchilla’s environment and the conventional farm setting raises important questions about welfare. In nature, chinchillas spend much of their time hidden in tight crevices and burrows, suggesting a strong instinct for enclosed, secure spaces [3]. However, this behavior does not necessarily imply a true preference for small, barren cages in captivity. Scientific studies on the effects of cage size on chinchilla behavior and welfare are still scarce. Among the few available, notable examples include Łapiński et al. [16] and Szendrő et al. [17]. Łapiński et al. [16] investigated how enclosure size and complexity affect the behavior of farmed adult female chinchillas, while Szendrő et al. [17] analyzed adult chinchillas’ preferences for cages of different sizes. However, both studies focused on fully mature animals, omitting the preferences of young chinchillas during the critical period from weaning to sexual maturity. This developmental stage may involve specific behavioral and environmental needs different from those of adults, underlining the importance of dedicated studies on younger age groups.

The aim of the present study was to examine how the cage dimensions influence the housing preferences of juvenile chinchillas. In particular, we tested whether young chinchillas prefer a larger versus a smaller cage floor, a higher versus a lower cage, or a combination of increased floor area and height. Understanding these preferences will provide insight into the spatial needs of chinchillas and inform evidence-based recommendations for improving housing design and welfare standards in chinchilla farming.

## 2. Materials and Methods

### 2.1. Animals and Housing Conditions

A total of 123 juvenile standard chinchillas (*Chinchilla lanigera*) were used in this study. All animals were healthy and had been housed under standard farm conditions prior to the experiment. The juveniles were obtained from a commercial breeding farm and were approximately 3–4 months old at the start of the trials (weaned but not yet sexually mature).

The experiment was conducted using a group housing system, with three chinchillas randomly assigned to each cage block. Neither sex nor body weight was used as an experimental variable; animals were selected based on phenotypic assessment, confirming appropriate size for age, good general condition, and overall health.

During the experiment, chinchillas were housed in an indoor facility with controlled microclimate conditions similar to their original housing. Ambient temperature was maintained at 19 °C, with relative humidity around 65%, replicating typical farm conditions. A fixed light–dark cycle was provided, with artificial lighting for 9 h per day (approximately 07:00–16:00) and darkness for the remaining 15 h, corresponding to the animals’ normal photoperiod. Throughout the study, the chinchillas had free access to a commercial pelleted diet (containing 19% crude protein, 12% crude fiber, and 11.3 MJ/kg digestible energy), and fresh water was provided ad libitum. In each cage, a similar feeder and drinker were installed. No environmental enrichments (such as shelters, platforms, nesting material, sand bathing bowls, or chewing blocks) were placed in the cages during the trials to avoid influencing cage preference behaviors.

### 2.2. Enclosure Configurations and Experimental Design

The experiment was designed as a series of preference tests in which juvenile chinchillas could freely move between connected cages of different sizes. In each specially constructed cage block, three animals were housed together, and they could choose among the interconnected cages (i.e., 0, 1, 2, or 3 chinchillas could stay in any given compartment at a time). The cage blocks were made of wire mesh (including mesh flooring) and had small open passageways in the side walls, allowing the animals to move freely between compartments.

**Figure 1:**
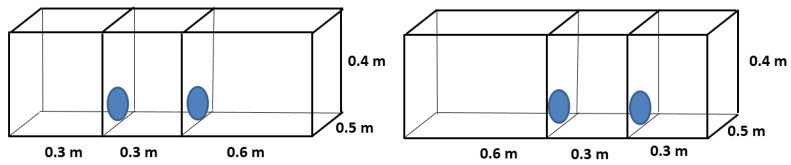
Design of cage blocks for preference testing of young chinchillas, comparing 0.4 m high cages with different floor areas (0.15 and 0.3 m^2^).

**Figure 2:**
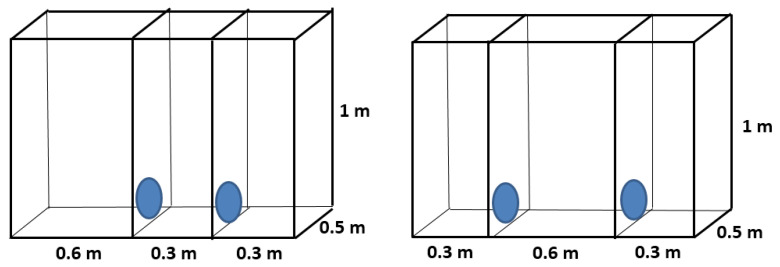
Design of cage blocks for preference testing of young chinchillas, comparing 1 m high cages with different floor areas (0.15 and 0.3 m^2^).

**Figure 3:**
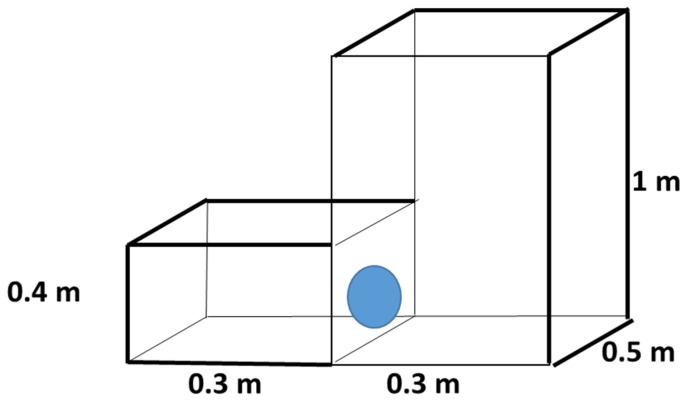
Design of cage blocks for preference testing of young chinchillas, comparing cages with different heights and a 0.15 m^2^ floor area.

**Figure 4:**
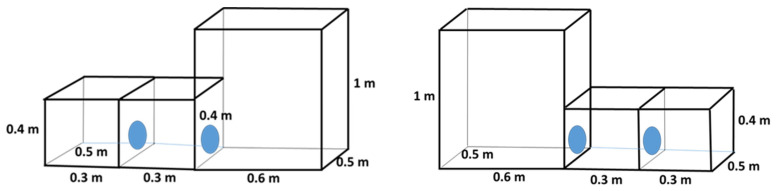
Design of cage blocks for preference testing of young chinchillas, comparing small and low, and large and high cages.

Three types of cage size comparisons were evaluated in separate trials:

(1) Floor Area Preference: To examine floor area preference, chinchillas were offered a choice between one large cage and two small cages, with cage height kept constant. Each small cage had a floor area of 0.15 m^2^, while the large cage had a floor area of 0.30 m^2^. Two small cages were used instead of one to ensure that the total available floor area matched that of the large cage (2 × 0.15 m^2^ = 0.30 m^2^), while still providing a simultaneous and spatially distinct choice. Two separate sets of trials were conducted at 0.4 m cage height (Figure 1) or at 1.0 m cage height (Figure 2) to examine whether vertical space availability altered floor area preferences. In each trial, the chinchillas could move at will between the one large cage and the two small cages through the connecting openings.

(2) Height Preference: Chinchillas were offered a choice between a low cage and a high cage, while keeping the floor area constant. One cage was 0.4 m in height (low) and the other was 1.0 m in height (high). Both cages had an identical floor area of 0.15 m^2^, so only the vertical space differed (Figure 3). Chinchillas could freely move between the low and high cages through the wall opening during these trials.

(3) Combined Size Preference: To evaluate the combined effect of floor area and height, the chinchillas had a choice between a small–low cage and a large–high cage. In this configuration, one cage measured 0.15 m^2^ in floor area and 0.4 m in height, whereas the other cage measured 0.30 m^2^ in floor area and 1.0 m in height (the larger and higher enclosure) (Figure 4). This comparison tested whether simultaneously increasing both horizontal and vertical space would alter the animals’ preferences.

In all the above comparisons, only cage dimensions were varied; all other factors (feeder and water bottle placement, flooring type, etc.) were identical between the cages. Each chinchilla participated in only one type of comparison (floor area, height, or combined) to ensure independent observations. Each trial involved 30–33 chinchillas, with 10–11 replicate runs of 5 days each.

### 2.3. Behavioral Observations and Data Recording

Behavioral monitoring began immediately after the 24 h acclimation period. Each preference trial lasted for 5 consecutive days per animal. An infrared camera was positioned in front of each cage block to continuously record movements 24 h per day without disturbing the animals (infrared illumination allowed unobtrusive monitoring in darkness when chinchillas are most active).

A scan-sampling method was employed to quantify enclosure preference: the position of each of the 3 chinchillas was noted every 30 min, yielding 48 observations per animal per day. Over the 5-day trial for a given animal, a total of 240 location data points were collected. These observations were later aggregated to determine the proportion of time each chinchilla spent in each type of enclosure. To assess daily patterns, timestamps were categorized into “light” (07:00–16:00, resting phase) and “dark” (16:00–07:00, active phase) periods, which allowed analysis of whether enclosure preference differed between the typical rest period (daytime) and active period (night). All video files were reviewed by the experienced researchers, and location data were logged manually. Determining which cage an animal occupied was straightforward on the video; although observers were aware of the enclosure types, the clear differentiation between cages meant there was no risk of observer bias in recording. All chinchillas remained in good health during the trials, and their behavior (e.g., feeding, grooming, resting) appeared normal.

### 2.4. Statistical Analysis

For each comparison, the number of observations in each type of enclosure (e.g., small vs. large, or low vs. high, or small–low vs. large–high) was summed for each chinchilla over its 5-day trial. These counts were used to calculate preference percentages and to perform hypothesis testing. The primary analysis was a chi-square (χ^2^) test to evaluate whether the observed distribution of cage use differed significantly from an expected equal distribution (i.e., 50:50 use of each option). Chi-square tests were conducted separately for each type of enclosure comparison. In addition, within each trial, we examined whether there were any systematic changes in preference over the 5 days (e.g., habituation effects) or differences between the light and dark periods. Day-to-day consistency in cage choice was checked by comparing daily counts; since no significant day-to-day variation was detected, data were pooled across the 5-day period for each animal. Differences between distributions in light and dark periods of day were assessed descriptively and via subgroup analyses, but this was not the primary focus of statistical analysis. All analyses were carried out using IBM SPSS Statistics (Version 10.0; SPSS Inc., Chicago, IL, USA). Results were considered statistically significant at *p* < 0.05, with highly significant differences noted at *p* < 0.001.

### 2.5. Animal Welfare and Ethics

This study involved only non-invasive behavioral observations (preference tests) and did not include any procedures causing pain, suffering, distress, or lasting harm to the animals. Therefore, formal approval from an Ethics Committee was not required, in accordance with Article 1, Paragraph 5(a) and (f) of European Council Directive 2010/63/EU, which exempts observational studies without intervention. All procedures were carried out following the Directive’s general principles, including minimizing animal stress and ensuring appropriate housing and care. No harm came to the animals as a result of the study, and all were returned to their original housing system in good health after the experiments.

## 3. Results

There are three basic ways to increase the size of a cage: by increasing its floor area, by increasing its height, or both. We accordingly conducted three sets of preference tests. First, we examined chinchilla preferences when the cage floor area was doubled (small vs. large cage). Next, we tested preferences when the floor area was unchanged, but the cage height was increased (low vs. high cage). Finally, we observed the chinchillas’ choices when both floor area and height were increased together (small–low vs. large–high cage).

### 3.1. Cage Blocks with Different Floor Area

Because the three juveniles tended to huddle together, especially during resting/sleeping periods, they were often all found in the same compartment at a given scan. This meant that at any scan, between 0 and 3 chinchillas could be present in either the large cage or the small cages combined. In practice, the juveniles showed a strong tendency to cohabit in one of the small cages during the day.

#### 3.1.1. Cage Height 0.4 m (Low Cage Block)

When cage height was 0.4 m for all sections, the small cages were chosen about twice as often as the large cages on average (approximately 66% vs. 34% of observations; *p* < 0.001). A significant effect of day was observed: the preference for the small cages increased from 52% on day 1 to 76% on day 5 (and conversely, large cage use declined from 48% to 24% over that period), indicating that the bias toward the small cages became stronger after several days.

**Figure 5:**
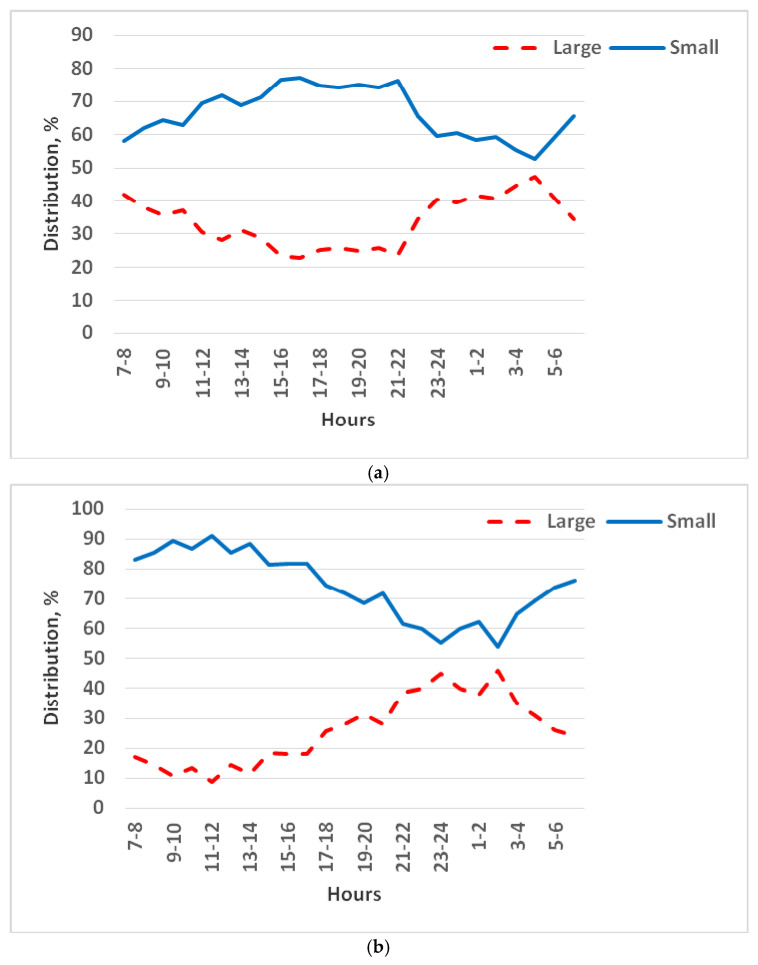
Distribution of three juvenile chinchillas’ location preferences between small (0.15 m^2^) and large (0.30 m^2^) cages at two heights: (**a**) 0.4 m and (**b**) 1.0 m. Percentages indicate the proportion of animals observed in each cage type during 24 h monitoring.

Differences between cages were more pronounced during the resting phase (light period) than during the active phase (dark period). However, at both periods of day, the small cages were used roughly twice as much as the large cages (*p* < 0.001). In the light period, chinchillas spent about 68% of observations in the small cages vs. 32% in the large, and in the dark period, 65% in small vs. 35% in large. The small cage preference peaked (75%) in the late afternoon/evening hours (15:00–22:00) and was lowest (53–60%) in the late night/early morning hours (23:00–06:00) (Figure 5a). In general, the presence of chinchillas in small cages increased during the morning (07:00–17:00) and decreased during the late night (21:00–05:00).

#### 3.1.2. Cage Height 1.0 m (High Cage Block)

When the cage height was 1.0 m, the juveniles still showed a marked preference for the small cages. No clear day-to-day trend was observed in this case; the preference remained fairly stable, averaging about 75% of observations in the small cages vs. 25% in the large cage across days 1–5 (*p* < 0.001).

There were significant differences between the cage preferences of animals in the light and dark periods. During the daytime resting period, the chinchillas stayed in the small cages about six times more often than in the large cage (86% of observations in small vs. 14% in large). At night, this difference was smaller (approximately a twofold difference: 67% small vs. 33% large). The highest frequency of small cage use (85–90%) occurred in the morning hours (09:00–14:00), after which it declined to 60% around midnight, and then increased again in the early morning after 03:00 (Figure 5b).

### 3.2. Cage Blocks with Different Heights

When chinchillas had a choice between a low cage (0.4 m high) and a high cage (1.0 m high) with the same floor area (0.15 m^2^), they preferred the lower cage. The juveniles spent more than twice as much time in the low cage as in the high cage (approximately 70% vs. 30% of observations; *p* < 0.001). This preference remained fairly consistent across the 5-day observation period (no significant change over days 1–5).

**Figure 6:**
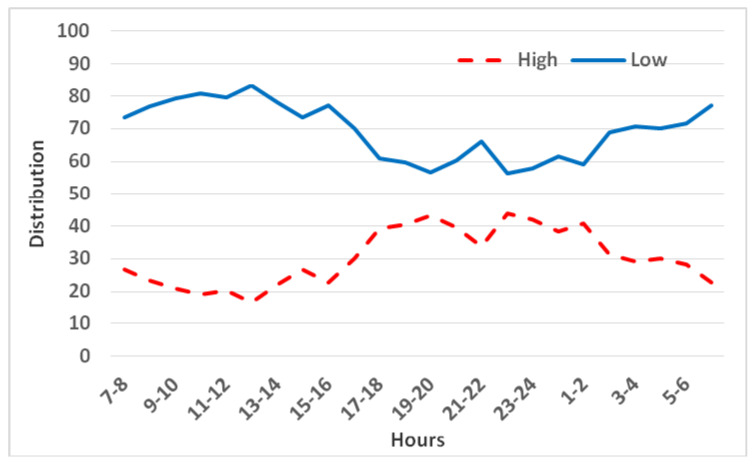
Distribution of three juvenile chinchillas’ preferences between low (0.4 m) and high (1.0 m) cages with identical floor area (0.15 m^2^).

As in the floor area trials, the preference was somewhat stronger during the day. The chinchillas spent about 78% of daylight observations in the low cage compared to 64% at night. The largest difference in low vs. high cage use was observed around midday (67% more in the low cage at that time). From roughly 03:00 to 17:00, the juveniles were found in the low cage in 70–80% of observations, whereas between 17:00 and 02:00, the low cage use dropped to around 60% (Figure 6). Thus, even during the active phase, the low cage was generally favored.

### 3.3. Cage Blocks with Different Floor Size and Height (Combined)

After examining floor area and height separately, we also evaluated preferences when both dimensions of the cage were changed. In this combined test, chinchillas could choose between a small–low cage (0.15 m^2^ × 0.4 m) and a large–high cage (0.30 m^2^ × 1.0 m).

The juveniles exhibited large differences in preference between the two options. Overall, the young chinchillas were observed about four times as often in the small–low cage (79% of observations) as in the large–high cage (21%; *p* < 0.001). This strong preference persisted throughout the 5-day period, with no significant change in the proportion of time spent in each cage from day 1 to day 5 (daily averages remained in the 77–80% vs. 20–23% range).

**Figure 7:**
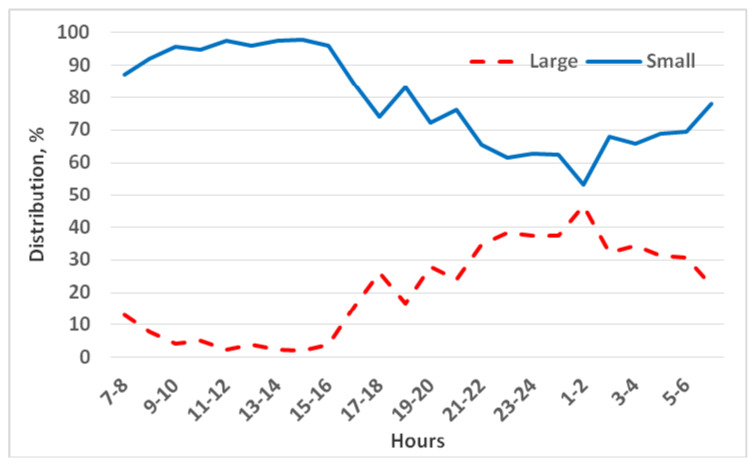
Distribution of three juvenile chinchillas’ preferences between a small–low cage (0.15 m^2^ × 0.4 m) and a large–high cage (0.30 m^2^ × 1.0 m).

During the light period, chinchillas almost exclusively stayed in the small–low cage (95%) (Figure 7). Thus, when resting, the juveniles virtually never chose the large cage. In the active (dark) period, a significant difference between cage types was still observed: the chinchillas were found about twice as frequently in the small–low cages (69% of nighttime observations) as in the large–high cages (31%). The preference gap narrowed at certain times during the night, but at no point did use of the large cage exceed that of the small cage. Even during the night, the small–low cage was generally favored by 20–40% more observations than the large–high cage. In summary, juveniles overwhelmingly chose the smaller, lower enclosure over the larger, higher one, especially during the lighting hours.

## 4. Discussion

Our findings demonstrate a strong propensity for chinchillas to choose smaller, more enclosed spaces rather than larger ones. Across all comparisons, the juveniles spent significantly more time in the smaller or lower cage compartments than in the more spacious alternatives. In the floor area trials, for instance, young chinchillas chose the small cages about 2–3 times more frequently than the large cage (independently of the cage height). In the height-only trials, chinchillas chose the low cage over the high cage by a factor of 2.3. During the resting (light) period, these differences were even more pronounced: in the floor area test at 0.4 m height, juveniles used the small cages 2.7 times more often than the large cage (and 2.1 times more often at 1.0 m height). In the height test, they used the low cage 3.5–4.3 times more often than the high cage during daylight, versus 1.6–1.8 times at night. These results demonstrate that chinchillas show a high preference for staying in smaller, lower cages rather than in larger or taller ones, largely independent of time of day or the age of the animals. Even adult chinchillas in similar tests preferred smaller cages by a wide margin [16,17].

It is informative to compare our results with similar preference studies in other species, particularly rabbits and laboratory rodents, which have somewhat analogous ecology. Rabbits, like chinchillas, are prey animals that in nature spend daylight hours in burrow systems (warrens) and emerge at dusk to forage. Several studies have examined enclosure size preferences in rabbits. Mikó et al. [18] found that domestic rabbit does initially spent significantly more time in a smaller cage when given a choice between a standard and double-sized cage, though this difference diminished after the first day as the rabbits grew more accustomed to both spaces. During the resting (light) period, the rabbit does in that study preferred the smaller compartment, whereas at night, their cage use became more balanced. Matics et al. [19] observed that immediately after weaning (at 3 weeks of age), rabbit kits overwhelmingly occupied the smallest available cage when offered multiple cage sizes, and only later (by 7 weeks of age) did their distribution equalize across space. These patterns in rabbits—a transient early preference for smaller spaces that fades over time or with familiarity—are qualitatively similar to our observation in chinchillas. However, in this case, the juvenile chinchillas’ preference for smaller cages remained strong throughout the trial.

Despite progressive improvements in mandated cage sizes for laboratory rodents [20], research has shown that larger cages do not always confer obvious welfare benefits in practice. For example, Sharp et al. [21] found that housing adult male rats in smaller cages (with less floor space) reduced their resting heart rate and blood pressure, and led to slightly greater weight gain, compared to rats in larger cages—all without any increase in feed intake. These observations suggest that the rats were possibly less stressed in the smaller, more confined environment. Similarly, Barker et al. [22] observed that increasing cage surface area for group-housed rats encouraged more anxiety-like behaviors in subordinate individuals, implying that simply providing more space per rat did not improve welfare and might even exacerbate social stress. Bailoo et al. [23] also reported that space allowance had little impact on several welfare measures in mice; for instance, fecal glucocorticoid metabolite levels were not affected by increased floor area. On the other hand, if the cage floor area is held constant while group size differs, higher stocking density can negatively influence behavior. Gaskill and Pritchett-Corning [24] found that mice in the highest-density cages (small cages with the same number of mice) showed reduced playing behavior in pups and more frequent stress posture in adults, compared to those in larger cages with lower density. In general, results from rabbits and rodents suggest that simply using a larger floor area does not straightforwardly equate to better welfare or preference fulfillment; the context (including social grouping and enrichment) matters.

In the present chinchilla trials, providing more floor space likewise did not lead to greater use of that space—the juveniles consistently favored the smaller area. The differences between small and large cage usage were even more pronounced for chinchillas than the modest differences seen in some rabbit studies, and we found no evidence that the extra space provided any detectable benefit from the animals’ perspective (as measured by voluntary use). In the floor area tests, the chinchillas did not gradually shift toward using the large cage more over time; if anything, they used it less over successive days (especially in the low 0.4 m configuration) as they increasingly preferred the small cages. Thus, the presumed advantage of a larger cage could not be demonstrated in this preference paradigm.

Concerning cage height, our results showed that chinchillas strongly preferred lower cages over taller ones (when the floor area was equal). The juveniles spent 69–75% of their time in the low (0.4 m) cages versus only 25–31% in the high (1.0 m) cages. Comparing periods of the day, the difference was larger in daylight (around 78–81% low vs. 19–22% high) than at night (roughly 62–64% low vs. 36–38% high). These findings align with studies on rabbits that show a low preference for fully open or very tall spaces. Growing and adult rabbits do not prefer completely open-top pens; in a preference test where growing rabbits could choose among cages of 20, 30, or 40 cm height or an open-top enclosure, the open-top was used only about half as much as any of the enclosed cages (15% vs. 27–30%) [25]. During the rest period, rabbits most often chose the lowest cage (20 cm high). When elevated platforms were provided in pens [26], most rabbits chose to rest under the platform (a “shelter” area), indicating a preference for a covered space within a larger environment. Even for adult rabbits offered cages of 30, 40, or 50 cm height or open-top, the distribution of time was 26%, 31%, 32%, and only 11% in the open-top, respectively. These comparisons underscore the importance of a perceived “ceiling” or refuge.

In the combined floor area and height comparison in the present study, the differences in preference were the most marked. Juvenile chinchillas spent on average 79% of their time in the small–low cage across the day. During the daylight hours, they rarely entered the large–high cage (only 5% of observations); essentially all resting occurred in the small–low enclosure. During the active night hours, their use of the two cages was more balanced (about 69% small vs. 31% large) but still significantly favored the small–low cage. We noticed that there were periods at night when the difference between small and large cage use narrowed (the juveniles would occasionally venture into the large cage during certain hours), but these periods were relatively short for the young animals. By contrast, adult chinchillas in the same test exhibited a longer period at night when the usage of the small vs. large cage was nearly equal [17], suggesting adults might explore the larger space a bit more. Nonetheless, both age groups strongly preferred the small–low cage overall. Taken together, our results indicate that, based on these preference tests, there is no apparent advantage (from the chinchillas’ perspective) to housing them in a larger cage than the traditional small cages they are accustomed to.

It should be noted that other measures of animal welfare and productivity in rabbits echo these preference outcomes. Studies comparing standard vs. larger cages for breeding rabbits have often found minimal impact on performance or well-being. Rommers and Meijerhof [27] found that doubling cage floor area (with constant height) or increasing cage height (with similar floor area) led to only minor differences in rabbit does productivity, with overall production being similar in standard and larger cages. Mirabito et al. [28] tested three cage sizes for rabbit does and observed no significant differences in reproductive performance or general behavior. Bignon et al. [29] likewise reported no difference between the reproductive performance of does in standard cages vs. larger cages, although does in larger cages were more active and spent less time lying down (i.e., they did use the space to move more). Interestingly, Prola et al. [30] found higher fecal corticosterone levels in rabbit does kept in smaller cages than in those in larger cages. This physiological indicator suggests that, at least for adult rabbits, a smaller enclosure might elevate stress—a nuance that preference tests alone might miss. Instead of simply increasing cage size, providing structural enrichments (like platforms) in rabbit cages has been recommended to improve welfare, as it offers greater mobility and opportunities for retreat without exposing the animals to open space, which they might find threatening [31].

Chinchillas, like rabbits, are prey animals. In the wild, European rabbits remain concealed in burrows (warrens) during the day, which serve as safe havens [32,33], and only emerge at dusk to forage nearby [34,35]. Chinchillas similarly seek out places where they feel safe. Our results indicate that farmed chinchillas retain the same fear-driven need for security as their wild ancestors; they consistently chose the more secure, enclosed space over a more exposed, larger area. This instinctual behavior explains why they preferred to stay in a smaller cage rather than a large one when given the option.

According to Baumans [36], providing structural complexity in an animal’s enclosure—such as hiding places, elevated platforms, and enrichment objects—may be more important for welfare than simply increasing the overall floor area. While a minimum amount of space is necessary, it is the quality and organization of the space that better support natural behaviors and psychological welfare. This view is supported by the EFSA Scientific Opinion [37], which emphasizes that chinchilla housing systems should enable species-specific behaviors, including hiding, climbing, gnawing, and exploration. Environments that lack these features are considered inadequate, as they fail to meet fundamental behavioral needs.

Although our study did not consider environmental enrichment and cannot directly evaluate its impact, the consistent preference by young chinchillas for smaller enclosures likely reflects a motivation to seek out safe places—a typical behavioral strategy of prey species. Importantly, this preference should not be interpreted as evidence that small or barren cages promote good welfare. As has been shown in studies of other species, animal preferences may reflect coping mechanisms in response to restricted environments, rather than ideal housing conditions.

Within the Five Domains model of animal welfare (nutrition, physical environment, health, behavior, and mental state), our results primarily reflect the animals’ search for a secure physical environment and their immediate mental state. However, small, barren cages can simultaneously compromise other welfare domains. A restricted space limits opportunities for movement and behavioral expression (behavior domain) and may contribute to health issues such as obesity or the emergence of abnormal repetitive behaviors like fur-chewing (health domain), which have been repeatedly observed under commercial farming conditions [38,39,40]. While genetic predispositions may play a role [41], the lack of environmental complexity and stimulation is widely considered a major contributing factor.

Therefore, instead of interpreting the preference for smaller spaces as justification for minimalistic housing, our findings highlight the need to design environments that simultaneously offer security and stimulation. Future studies should empirically investigate how chinchillas use complex environments—for instance, whether adding hiding structures to a large cage increases its appeal—and on measuring welfare outcomes (health, stress physiology, behavior) in enriched versus barren housing. This would allow for a more holistic evaluation of housing systems in line with the Five Domains model, ensuring that welfare improvements go beyond simple spatial parameters and address the animals’ full range of needs [37].

## 5. Conclusions

This study demonstrated that young chinchillas consistently spent more time in smaller cages, whether “small” was defined by floor area, cage height, or both, which are similar to the traditional cages used in chinchilla farming. Based on these results alone, the introduction of significantly larger cages for housing chinchillas is not supported by the animals’ observed preferences. Simply providing more space did not encourage juveniles to utilize it; on the contrary, they gravitated to the smaller compartments. However, these findings should be interpreted with caution. Further research is needed to clarify the underlying reasons for chinchillas’ small-space preference and to determine how to best balance security and stimulation in their housing to optimize welfare. The preference for small enclosures likely reflects the chinchillas’ need for a secure, sheltered area rather than an absolute requirement for minimal space. Therefore, research and improvements in the housing of chinchillas should focus on designs that provide security (e.g., hiding places) within more enriched cages.

## Data Availability

The original contributions presented in this study are included in the article.
